# Bacterial Phenotype Variants in Group B Streptococcal Toxic Shock Syndrome^1^

**DOI:** 10.3201/eid1502.080990

**Published:** 2009-02

**Authors:** Parham Sendi, Linda Johansson, Samira Dahesh, Nina M. Van Sorge, Jessica Darenberg, Mari Norgren, Jan Sjölin, Victor Nizet, Anna Norrby-Teglund

**Affiliations:** Karolinska Institutet, Stockholm, Sweden (P. Sendi, L. Johansson, A. Norrby-Teglund); Basel University Medical Clinic, Liestal, Switzerland (P. Sendi); University of California San Diego, La Jolla, California, USA (S. Dahesh, N.M. Van Sorge, V. Nizet); Swedish Institute for Infectious Disease Control, Stockholm (J. Darenberg); Umeå University, Umeå, Sweden (M. Norgren); Uppsala University, Uppsala, Sweden (J. Sjölin); 2 Current affiliation: Clinic for Infectious Diseases, University Hospital Bern, Bern, Switzerland.

**Keywords:** Group B streptococci, toxic shock syndrome, carotenoid pigment, beta-hemolysin, research

## Abstract

We conducted genetic and functional analyses of isolates from a patient with group B streptococcal (GBS) necrotizing fasciitis and toxic shock syndrome. Tissue cultures simultaneously showed colonies with high hemolysis (HH) and low hemolysis (LH). Conversely, the HH and LH variants exhibited low capsule (LC) and high capsule (HC) expression, respectively. Molecular analysis demonstrated that the 2 GBS variants were of the same clonal origin. Genetic analysis found a 3-bp deletion in the *covR* gene of the HH/LC variant. Functionally, this isolate was associated with an increased growth rate in vitro and with higher interleukin-8 induction. However, in whole blood, opsonophagocytic and intracellular killing assays, the LH/HC phenotype demonstrated higher resistance to host phagocytic killing. In a murine model, LH/HC resulted in higher levels of bacteremia and increased host mortality rate. These findings demonstrate differences in GBS isolates of the same clonal origin but varying phenotypes.

## Abstract 2

Variants with markedly different expression of virulence factors can arise in invasive infection in humans.

Group B streptococci (GBS) are a major cause of sepsis in neonates and pregnant women. The incidence of invasive GBS disease in nonpregnant adults is growing, in particular in elderly persons and in those with chronic underlying conditions (e.g., diabetes mellitus) (*1*). Recently, cases of the severe, life-threatening syndromes of necrotizing fasciitis (*1*) and toxic shock syndrome due to GBS have been reported in neonates (*2*) and immunocompromised persons (*3*), reminiscent of a disease course more commonly associated with group A streptococci or *Staphylococcus aureus*. We report a case of GBS necrotizing fasciitis and toxic shock syndrome in a previously healthy person. We discovered 2 specific phenotypic variants of the bacterium from the tissue site of infection. Genetic and functional analysis of these variants provides insight into the potential contribution of specific bacterial virulence factors to these emerging GBS clinical syndromes.

Of GBS virulence factors, 2 of the best characterized are its exopolysaccharide capsule and the surface-associated toxin, β-hemolysin/cytolysin (β-h/c). The capsule contributes to immune resistance by inhibiting complement deposition and activation on the bacterial surface, thereby reducing opsonophagocytic clearance (*4*). GBS production of β-h/c is encoded by the genes of the *cyl* operon (*5*,*6*) and is associated with direct lysis of a variety of eukaryotic cell types (*7*–*9*), inflammatory activation (*10*–*12*), and virulence in animal models (*10*,*13*,*14*). GBS β-h/c expression is linked to expression of an orange pigment with antioxidant properties (*6*,*15*), and these 2 factors act in concert to impair macrophage-based immune clearance (*16*).

In the traditional clinical view of invasive GBS pathogenesis, a bacterial isolate enters a normally sterile site from a focus of mucosal colonization or recent acquisition of the pathogen. However, as the present case will illustrate, selective pressures in vivo may cause differential expression of certain GBS surface components during colonization or dissemination. Thus, in vivo pathogenesis from a bacterial perspective is likely more dynamic.

## Case Report

A previously healthy 50-year-old man was admitted to the hospital with fever, severe pain and swelling of the right shoulder and arm, 1 week after moderate trauma. The extremity was erythematous, markedly swollen, and intensely tender. In the emergency department, the man’s condition rapidly deteriorated to septic shock. After receiving immediate support with oxygen, intravenous fluids, and antimicrobial agents (penicillin G, 3 g, 4×/day, and a single dose of 120 mg gentamicin), he was transferred to the intensive care unit. In addition to mechanical ventilation and vasopressors, medical treatment included intravenous immunoglobulins and corticosteroids. Because necrotizing fasciitis was suspected, wide debridement was performed, which confirmed the clinical diagnosis. In tissue specimens obtained during the operation, gram-positive cocci with typical streptococcal morphologic features were abundant. After samples underwent overnight culture on blood agar plates and the organisms were identified to species level, GBS was isolated. This pathogen also grew in cultures of blood obtained while the patient was in the emergency room. Antimicrobial drug treatment was changed to the combination of clindamycin and penicillin G. After a total of 3 repeated debridements and antimicrobial drug treatment for 6 weeks, the outcome was favorable. At follow-up after 6 months, the patient had only a slight radiating pain in the arm.

## Methods

### Sources of Bacteria

We included the following in specific, comparative assays: 2 serotype V GBS strains isolated from a colonized person (VK9) and a neonate with sepsis (CNCTC), GBS NEM316, and 1 group A streptococci serotype M1T1 isolate from a patient with streptococcal toxic shock syndrome (isolate 5448).

### CAMP Test, Serotyping, Antimicrobial Susceptibility, and Pulsed-Field Gel Electrophoresis

Identification of the isolates was confirmed by CAMP testing, and serotype determination was achieved by using a coagglutination typing kit (Essum, Bacterium AB, Umeå, Sweden). Antimicrobial susceptibility and MICs were determined by the Kirby-Bauer disk diffusion method and by Etest (AB Biodisk, Solna, Sweden), respectively. GBS isolates were subjected to pulsed-field gel electrophoresis (PFGE) by using the restriction enzymes *Sma*I or *Xma*I (New England Biolabs, Ipswich, MA, USA) or *Apa*I (Promega, Madison, WI, USA), as described (*17*).

### Measurement of Pigment and Hemolytic Activity

Pigment was extracted as described (*16*). The optical density (OD) of the pigment extracts was measured at a dilution of 1:4 in a spectrophotometer (WPA Biowave, Biochrom, Cambridge, UK). The hemolytic activity was determined by measuring hemoglobin release in the supernatant (by OD), after pigment extracts were incubated with an equal volume of 1% sheep erythrocytes for 1 h. Phosphate-buffered saline (PBS), glucose alone, and erythrocytes lysed with 0.1% sodium dodecyl sulfate (SDS) were used as negative and positive controls, respectively. The results were related to SDS (100%) and expressed as hemolytic capacity. The hemolytic titer was assessed by a microtiter dilution method, as described previously (*7*).

### Capsule Expression

Buoyance density of overnight bacterial cultures was determined by Percoll gradient centrifugation as described previously (*18*). To analyze surface sialic acid expression, strains were grown to mid log phase, washed, and resuspended in PBS to an OD of 0.4. Sialic acids were hydrolyzed with mild acid, then filtrated, neutralized, and derivatized as previously described (*19*) for quantitative analysis by high-performance liquid chromatography. To visualize capsule expression, we incubated isolates on blood agar plates and in Todd-Hewitt broth (THB) overnight. Isolates were then washed and fixed with Karnovsky solution. After polymerization, samples were sectioned with an ultramicrotome (Reichert-Jung Ultracut E, Leica, Wetzlar, Germany), and analyzed by transmission electron microscopy (FEI, Philips, Morgani 268D, Aachen, Germany).

### Genetic Analysis

PCR was used to amplify the *cylA*, *cylE*, *covR*, *covS*, *rovS,* and *stk1* genes, as described previously (*6*), and sequences of amplicons were compared with the published genome of GBS serotype Ia strain A909 (*20*). Sequence alignment for all the genes showed >99% identity among GBS strains of different serotypes.

For heterologous expression of *covR/S*, the region was amplified by PCR from the GBS genome by using the forward primer 5′-GCGTCTAGAGAATAAGAAGGTTGGTGTAGATGGG-3′ and reverse primer 5′-CGCGGATTCGAAGCGCCTCTCTTATCACCTC-3′. The 2,286-bp amplicon was captured in pTTOPO according to the manufacturer’s instructions (Invitrogen, Carlsbad, CA, USA), then subcloned into expression vector pDCerm (*21*). The resulting pDC-CovRS plasmid was introduced into GBS by electroporation (*22*). Transformants were identified by erythromycin resistance, and plasmid presence was confirmed by PCR.

### RNA Isolation and Reverse Transcription–PCR

Total bacterial RNA was extracted from overnight cultures of GBS by using an RNeasy Mini Kit (QIAGEN, Hilden, Germany) per manufacturer’s instructions, except that bacteria were mechanically disrupted by using tubes with glass beads (Lysing Matrix B, MP Biomedicals, Solon, OH, USA). RNA samples were DNase treated (Turbo DNA-free; Ambion, Austin, TX, USA) to remove any contaminating DNA. One microgram of RNA was reverse transcribed to cDNA (Superscript First-Strand Synthesis Kit; Invitrogen) and used for PCR amplification with the following primer sets: *cfb* forward 5′-CTGGAACTCTAGTGGCTGGTG-3′ and *cfb* reverse 5′-CCATTTGCTGGGCTTGATT-3′; *cylK* forward 5′-ATTTATCTGGCGATCGGTTG-3′ and *cylK* reverse 5′-CCTTTGGCAAACCAATTAAATAAC-3′; *cylE* forward 5′-GTCGTA GTGGACAGGCAATCAC-3′ and *cylE* reverse 5′-CGAAATGATCGACAATGCAG-3′; *cpsG* forward 5′-CATGAACAGCAGTTCAACCG-3′ and *cpsG* reverse 5′-CTGACATAAACGTCGCTGGAC-3′; and *gyrA* forward 5′-CTTGGTGATGGGACGTTCAGG-3′ and *gyrA* reverse 5′-GCTGAAGCAGCACGACGAAC-3′. PCR mixtures contained primers at a concentration 1 μM and PCR mix (Supermix; Invitrogen) in a volume of 15 μL. Samples that had been prepared without reverse transcriptase served as controls for DNA contamination. The PCR products were visualized by electrophoresis on a 1% agarose gel containing ethidium bromide.

### Measurement of Growth Dynamics and Phenotype Stability

Bacterial growth rates were determined in THB and THB plus 1.5% yeast extract (THB + Y) by OD_600_ determination and enumeration of CFUs. Measurements were performed in triplicate. To evaluate phenotype stability, we passaged each variant isolate on blood agar (7 passages) and in various media (3 passages), including THB, THB + Y, Granada, and chromogenic (Strepto B ID agar; bioMérieux SA, Marcy-l’Etoile, France).

### Cytokine Stimulation

Peripheral blood mononuclear cells (PBMCs) were isolated from blood of 5 healthy donors by Ficoll-Hypaque gradient centrifugation (Lymphoprep; Axis Shield PoC AS, Oslo, Norway). PBMCs were stimulated with live bacteria (multiplicity of infection [MOI] ≈1:1) for 2 h. Uninfected PBMCs served as negative controls. Interleukin (IL)-1β, IL-8, and tumor necrosis factor α (TNF-α) in cell culture supernatants were determined by Luminex multiplex assays (BioSource International, Camarillo, CA, USA) and the Luminex^100^ instrument (Luminex, Austin, TX, USA).

### Murine Model of GBS Infection

Male CD-1 mice (Charles River Laboratories, San Diego CA, USA) 6–8 weeks of age were injected intraperitoneally with 6–8 × 10^6^ (low inoculum) or 5–7 × 10^7^ CFU (high inoculum) of either GBS phenotype variant and monitored for survival. After 6 h, blood was collected by retro-orbital puncture and assessed for levels of bacteremia by serial dilution plating on blood agar plates. Ethics approval for animal experimentation was obtained from the Animal Care Program of the University of California, San Diego, CA, USA.

### Determinations of Bacterial Growth and Killing Rates in Human Whole Blood

Bacterial growth rates in freshly collected whole blood from 3 nonimmune human donors were determined by enumeration of CFUs. The blood was incubated with 10% volume of an overnight bacterial culture under mild agitation. Measurements were performed in duplicate.

For whole blood killing assays, inocula of 100 CFU in 100 μL were mixed with 300 μL human blood (n = 5 donors) in heparinized tubes and incubated for 1–3 h with mild agitation. Dilutions were plated on blood agar for enumeration of CFU. Autologous plasma from nonimmune donors were used as controls.

### Neutrophil Opsonophagocytic Killing Assays

Bacteria (CFUs ≈10^6^) grown as described above were incubated with 10% normal human serum (i.e., source of complement) for 10 min and then mixed with autologous neutrophils (MOI ≈1:1) from the 5 nonimmune donors. Neutrophils were isolated by density gradient centrifugation using Polymorphprep solution (Axis Shield PoC AS). Controls included samples containing heat-inactivated serum and neutrophils, serum without neutrophils, and PBS. Immediately before and after 60 min incubation, 100 μL of sample solutions were removed and plated on blood agar plates (*23*). To determine the kinetics of GBS survival within neutrophils, we pelleted and resuspended the cells in minimum essential medium supplemented with L-glutamine, 125 μg/mL gentamicin (GIBCO, Invitrogen) and 5 μg/mL penicillin G (*24*). After 30, 60, and 90 min, cells were lysed and CFU enumerated. Samples with only bacteria were used as a control for the bactericidal effects of antimicrobial drugs.

### Statistical Analysis

Groups were compared by using a nonparametric paired test (Wilcoxon signed-rank test) and Kaplan-Meier plot; p values <0.05 were considered significant. Statistical calculations were performed by using GraphPad Prism, Version 4.03 (Graph Software, San Diego, CA, USA).

## Results

### Pigment and Hemolytic Activity

**Figure 1:**
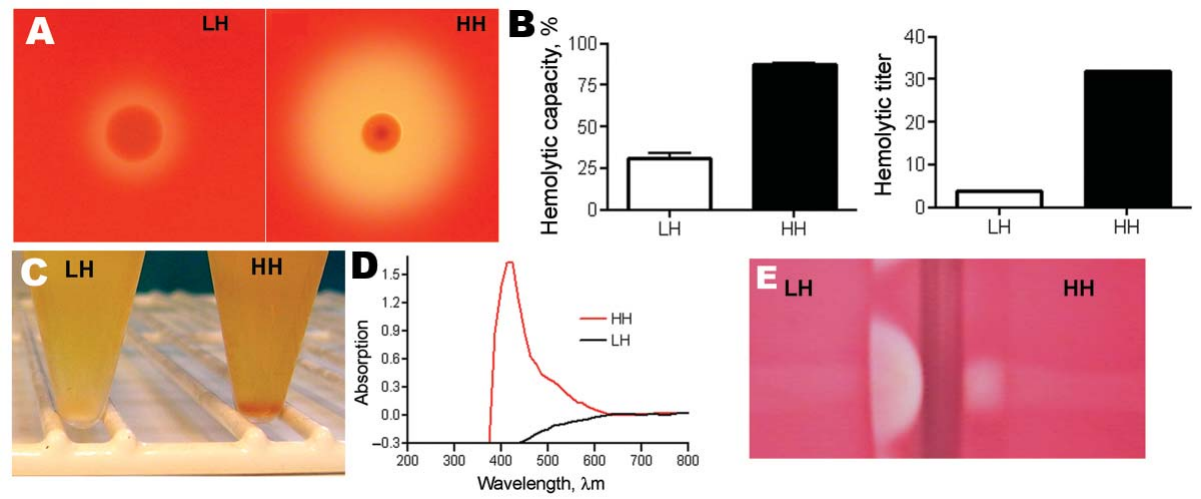
A) Hemolytic zone on blood agar plate after 48 h: Low hemolytic (LH) colony and high hemolytic (HH) colony. B) Hemolytic activity of the pigment extract presented as hemolytic capacity (left graph) relative to that of sodium dodecyl sulfate (100%) and as hemolytic titer (right graph) evaluated with a microdilution assay. Error bars indicate SEM. C) Phenotypic appearance of group B streptococci after overnight culture in Todd-Hewitt broth plus 1.5% yeast extract, displaying a white pellet (LH) and an orange pellet (HH). D) Absorbance profile of the pigment extract. E) Results of CAMP testing, which display a stronger reaction with the LH than with the HH phenotype.

Culture of a tissue sample on blood agar plates displayed GBS colonies with 2 different phenotypes, either high hemolytic (HH) or low hemolytic (LH) (Figure 1, panel A). The difference in hemolysis was corroborated by 2 different assays, which showed a 4- to 8-fold difference in hemolytic potential between the HH and LH variants (Figure 1, panel B). The HH phenotype had strong orange pigmentation; the pigmentation of the LH variant was difficult to detect in culture pellets or by spectrophotometric analysis (Figure 1, panels C and D). In CAMP testing, however, the HH phenotype displayed a weaker reaction than the LH variant (Figure 1, panel E). The distinct phenotypes of the 2 isolates proved to be stable after up to 7 passages in various media.

### Encapsulation

**Figure 2:**
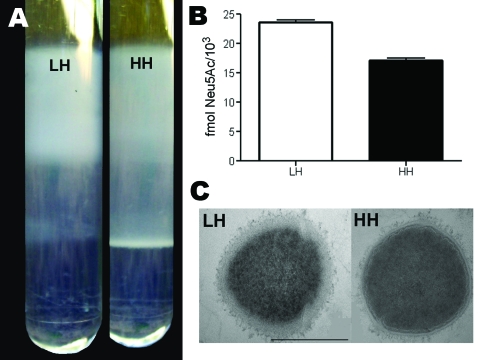
A) Buoyant density analysis of the low hemolytic (LH) and high hemolytic (HH) strains, exhibiting lower and higher buoyant density, respectively. B) Quantification of group B streptococci sialic acids expressed as fmol N-acetylneuraminic acid/1,000 CFUs of the LH and HH phenotypes. Error bars indicate SEM. C C) Transmission electron microscopy of LH and HH phenotypic variants.

Evaluation of encapsulation by buoyant density centrifugation showed high density for the HH variant (consistent with low encapsulation [LC]) and low density for the LH strain (consistent with high encapsulation [HC]) (Figure 2, panel A). This difference in encapsulation was further confirmed by direct quantification of sialic acid levels (Figure 2, panel B) and by imaging with transmission electron microscopy (Figure 2, panel C).

### Clonal Origin of HH/LC and LH/HC Phenotypic Variants

Both phenotypes belonged to capsular serotype Ib and showed equivalent patterns of antimicrobial drug susceptibility or resistance. PFGE showed identical banding patterns after 3 restriction enzymes were used, which indicates that the 2 phenotypes had the same clonal origin. To assess whether a genetic mutation could explain the observed phenotypic difference, we sequenced genes implicated in β-h/c production or regulation. Although amplification and sequencing of *cylE*, *cylA*, *covS*, *rovS,* and *stk1* genes showed no difference between the isolates, a variation in the *covR* gene was observed. In the HH/LC variant, the *covR* sequence contained a 3-bp deletion, which eliminates a valine that was encoded at position 31 (LH/HC = 20′-LELLHEGYDVVVETNGRE-37′ vs. HH/LC = 20′-LELLHEGYDVV_ETNGRE-36′). The published sequence of the serotype Ia genome strain A909 is identical to that of the LH/HC variant.

### Sequence Variation in *covR* and Phenotypic Variation

**Figure 3:**
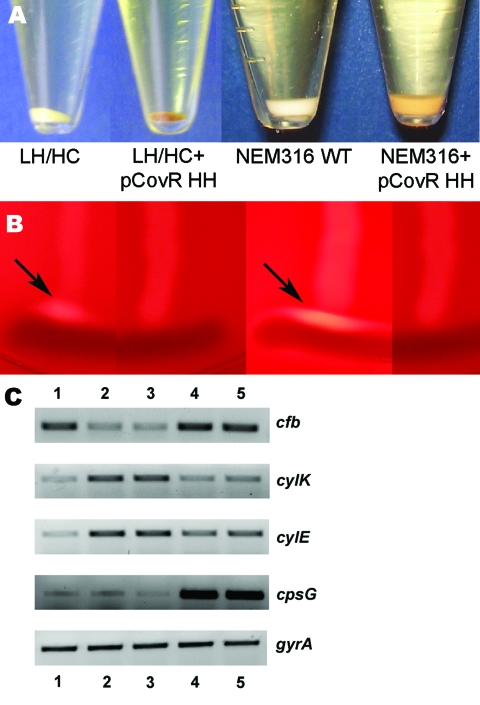
A) Difference in pigmentation of the low hemolytic (LH)/high encapsulation (HC) and NEM316 wild-type (WT) strains and their corresponding transformants expressing the *covR/S* locus of the high hemolytic (HH)/low encapsulation (LC) variant after overnight culture. B) CAMP testing with strains displayed according to panel A. The LH/HC variant and the NEM316 strain display a stronger reaction (arrows) than their corresponding transformants. C) Semiquantitative analysis of mRNA expression of CAMP factor (*cftb*), β-h/c (*cylK* and *cylE*) capsule (*cpsG*), and *gyrA* (housekeeping gene) using reverse transcription–PCR. Lane 1, LH/HC; lane 2, HH/LC; lane 3, LH/HC + pcovR HH; lane 4, NEM316 WT; lane 5, NEM316 + pcovR HH.

To confirm that the *covR* 3-bp deletion contributed to the observed phenotypic changes in the HH/LC strain, we expressed the *covR/S* locus from the HH/LC variant on an expression plasmid in the LH/HC variant and GBS strain NEM316. Introduction of the mutated *covR/S* locus transferred the phenotypic appearance of the HH/LC variant to both the LH/HC and NEM316 strains, which resulted in increased hemolytic activity with increased pigmentation (Figure 3, panel A) and decreased reaction in the CAMP testing (Figure 3, panel B). In addition, encapsulation, measured by mean production of sialic acid, was reduced in the LH/HC expressing the HH *covR/S* locus by 29% compared with the parent LH/HC strain (from 24 to 17 fmol N-acetylneuraminic acid/10^3^ CFU). To validate that the observed phenotypic changes were the result of changed transcriptional regulation, reverse transcription–PCR was performed on RNA isolated from the wild-type and transformant strains. The results demonstrated that introduction of the HH *covR/S* locus in either the LH/HC or NEM316 background reduced expression of the *cfb* gene (encoding CAMP factor) and increased expression of the *cyl* genes (Figure 3, panel C).

### Bacterial Growth Rate

**Figure 4:**
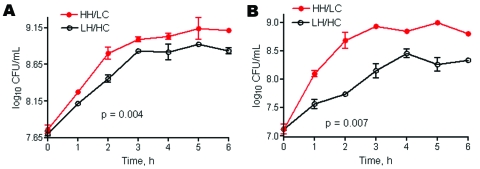
Growth curve of group B streptococcal variants, i.e., low hemolytic (LH)/high encapsulation (HC) and high hemolytic (HH)/low encapsulation (LC), in Todd-Hewitt broth plus 1.5% yeast cultured in a separate tube (A) or together in the same tube (B). Graph presented as mean + SD.

In culture media the HH/LC phenotypic variant grew markedly faster than the LH/HC variant (Figure 4, panel A) and even outgrew the LH/HC isolate when both phenotypes were cultured together (Figure 4, panel B). Both phenotypes showed a faster growth rate than the 2 control isolates (samples from vaginal colonization and neonatal sepsis patients), with division times of 35 and 45 minutes, respectively (data not shown).

### Cytokine Induction

**Figure 5:**
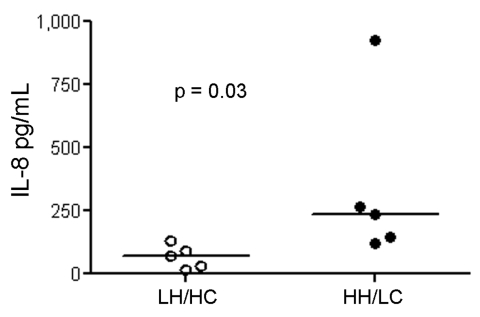
Interleukin (IL)– 8 induction in human peripheral blood mononuclear cells (PBMCs) (n = 5) using live bacteria. IL-8 concentration measured in cell culture supernatants of PBMCs were after exposure to live high hemolytic (HH)/low encapsulation (LC) and low hemolytic (LH)/high encapsulation (HC) bacteria. Horizontal lines indicate the median.

We further investigated the potential of the 2 variants to induce proinflammatory responses in human cells. Stimulation of PBMCs from different donors showed that both GBS variants induced IL-1β and TNF-α, but no overall difference was noted between the 2 isolates. Because β-h/c has previously been shown to be a potent inducer of IL-8 and β-h/c expression increases 4-fold in parallel with growth rate (*25*), we expected that a difference in IL-8 responses would be greatest when live HH/LC and LH/HC strains were used as stimuli. Indeed, live HH/LC bacteria induced significantly higher IL-8 levels than did the LH/HC isolate (p = 0.03) (Figure 5).

### Murine Toxic Shock Model

**Figure 6:**
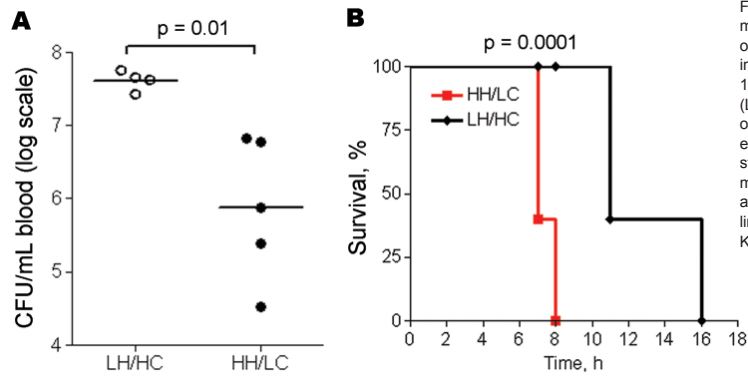
Murine toxic shock model (high inoculum). Groups of 5 mice were inoculated intraperitoneally with 5–7 × 10^7^ CFU of low hemolytic (LH)/high encapsulation (HC) or high hemolytic (HH)/low encapsulation (LC) group B streptococcal isolates per mouse. A) Level of bacteremia assessed after 6 h. Horizontal lines indicate the median. B) Kaplan-Meier survival plot.

Virulence of the 2 isolates was tested in a murine toxic shock model, by injecting each mouse intraperitoneally with 5–7 × 10^7^ CFU. Although all mice rapidly became bacteremic after inoculation, the bacterial load in blood was significantly higher in mice infected with the LH/HC strain (p = 0.01) (Figure 6, panel A). Nevertheless, the HH/LC bacteria caused death significantly earlier (p = 0.0001) (Figure 6, panel B).

### Resistance Toward Phagocytic Killing

**Figure 7:**
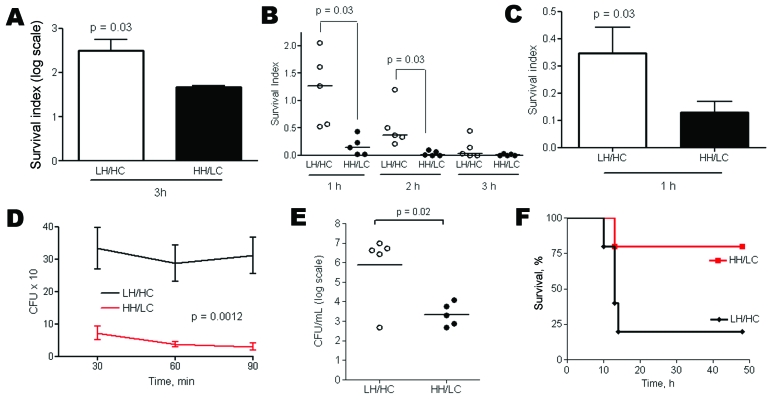
A) Human whole-blood killing assay after 3 h incubation, using 100 CFU bacteria in 100 μL phosphate-buffered saline (PBS) and 300 μL blood. Error bars indicate SEM. B) 100 CFU bacteria in 100 μL PBS and 1,000 μL blood. Survival index is calculated as follows: (CFU at the end of the assay)/(CFU at t = 0 h). Horizontal lines indicate the median. C) Opsonophagocytic killing assay after 1 h incubation, using a multiplicity of infection of ≈1:1 (CFU 10^6^/mL: neutrophils 10^6^/mL) and 10% volume serum. Error bars indicate SEM. D) Intracellular survival assays in neutrophils after 30, 60, and 90 min of extracellular antimicrobial drug exposure. Error bars indicate SEM. E) Murine model for invasive disease (low inoculum). Groups of 5 mice were inoculated intraperitoneally with 6–8 × 10^6^ CFU of low hemolytic (LH)/high encapsulation (HC) or high hemolytic (HH)/low encapsulation (LC) group B streptococcal isolates per mouse. Levels of bacteremia were assessed after 6 h. Horizontal lines indicate the median. F) Kaplan-Meier survival plot.

The higher bacterial load of the LH/HC phenotype in the mice in comparison to the previously noted higher growth rate by the HH/LC phenotype in media (Figure 4) suggested an increased resistance toward host immune defense in this phenotype. Indeed, in human whole blood, the LH/HC variant exhibited a higher growth rate than HH/LC (data not shown). We therefore further assessed these findings in several types of bactericidal assays. In a human whole-blood killing assay, the LH/HC phenotypic variant showed a higher survival index than the HH/LC variant at 2 different inocula (p = 0.03) (Figure 7, panels A and B). A similar survival advantage for the LH/HC variant against whole-blood killing was also observed in coculture assays in which both variants were used (data not shown). In an opsonophagocytic assay with purified neutrophils and complement, the LH/HC isolate again demonstrated a higher survival index than the HH/LC phenotype (p = 0.03) (Figure 7, panel C). The LH/HC strain also demonstrated greater intracellular survival within neutrophils compared with the HH/LC variant (p = 0.0012) (Figure 7, panel D).

We next sought to determine whether the enhanced resistance to bactericidal clearance would correspond to increased virulence in a low-dose infection model. Consistent with the high-dose sepsis model, recovery of bacteria from the bloodstream at 6 h postinfection was significantly higher in mice infected with LH/HC isolates than in those infected with the HH/LC phenotype (Figure 7, panel E). However, in contrast to high-dose challenge, in which death was accelerated in mice infected with the HH/LC variant, the increased resistance of the LH/HC against host phagocytic killing translated into sustained bacteremia and greater lethality (80% vs. 20%) in the lower dose infection model (Figure 7, panel F).

## Discussion

The rate of invasive GBS in nonpregnant adults is increasing, and most cases are found in elderly persons and those with underlying diseases (*26*). This study is based on a rare case of toxic shock syndrome and necrotizing fasciitis in an immunocompetent man without apparent risk factors. The GBS colonies obtained from the same tissue culture differed in phenotypic properties associated with 2 known GBS virulence factors, β-h/c cytotoxin and the exopolysaccharide capsule. A similar case was reported by Sigge et al. (*27*); they described a case of neonatal sepsis caused by GBS in which hemolytic and nonhemolytic colonies were displayed. Notably, only the hemolytic strain could be isolated from the maternal vaginal tract. The observation of GBS isolates of the same clonal origin, but with varying phenotypes, as described in our study and that of Sigge et al. (*27*) supports the concept of differential expression of certain virulence factors, either during the process of colonization or during infection of specific anatomic sites. These phenotypic changes may occur in response to selective pressures exerted by the host immune response, providing the pathogen a survival benefit. We therefore explored sequence differences in potential genetic clusters of this phenotypic variation and also investigated the functional differences between the 2 phenotypes. We demonstrated that the 2 isolates have distinct phenotypic characteristics but are of the same clonal origin. Moreover, our findings indicate that these variations in phenotypic appearance are associated with significant differences in resistance to host phagocytic killing and in the clinical course of experimental infection. Finally, we provide indications that this phenotype switch may occur due to a mutation in an important regulatory gene (*covR*).

In our clinical pair of phenotypic variants, pigment production and hemolytic activity was reduced (but not eliminated) in 1 variant. However, no mutations in the *cyl* genes, the operon encoding genes required for β-h/c production, were identified. Sequence analysis of several regulatory genes showed that the HH/LC phenotype contained a 3-bp deletion in the *covR* gene. Previous studies have shown that complete deletion of the GBS *covR/S* may result in up-regulation of *cyl* genes involved in β-h/c expression (i.e., hemolytic activity), down-regulation of genes in the GBS *cps* operon for capsule expression, reduced survival in serum, and reduced virulence in animals (*28**,**29*). Additionally, Δ*covR/S* mutants show reduced CAMP activity, increased adherence to epithelial cells, and increased β-galactosidase activity (*28*); these changes illustrate the influence of *covR/S* on multiple genes involved in phenotypic, virulence, and biochemical properties of GBS.

To explore whether the 3-bp deletion in *covR* harbored by the HH/LC mutant may contribute to the observed phenotypic variation between the 2 clonal isolates, we expressed the *covR/S* locus from the HH/LC in the LH/HC variant, as well as the NEM316 GBS genome strain. Indeed, overexpression of the *covR/S* HH locus in these 2 genetic backgrounds resulted in a phenotype switch, i.e., increased pigmentation associated with increased hemolytic activity, combined with decreased capsule production (as estimated by sialic acid quantitation) and CAMP reactivity. As demonstrated by reverse transcription–PCR, these observed phenotypic changes were paralleled by the expected changes in the mRNA transcripts for genes encoding β-h/c (*cylK*, *cylE*), CAMP factor (*cfb*), and *cps*G (an enzyme within the capsule biosynthetic operon).

Mutations in the related *covR/S* system of group A streptococci are induced under selective pressure of the innate immune system and contribute to the pathogenesis of invasive infection caused by strains of the M1T1 serotype, which is associated with necrotizing fasciitis and toxic shock syndrome (*30*,*31*). Future detailed genetic, transcriptional and mutational analysis of GBS invasive versus colonizing disease will be required to determine whether a similar paradigm exists in GBS.

Functional analyses of our clinical isolates showed that the HH/LC phenotype had a more rapid growth rate in culture media. Production of major GBS virulence factors (e.g., β-h/c, β-C protein) increases greatly with higher growth rate (*25*,*32*). In agreement with these results, the HH/LC phenotype induced a significantly higher release of the proinflammatory chemokine IL-8 than did the LH/HC phenotype. Considering the strong association between IL-8 serum levels and severity of sepsis (*33**,**34*), as well as the previously reported contribution of the β-h/c to the severe manifestations of septicemia in animal models (*13*,*35*–*37*), we compared the isolates in an in vivo sepsis model. Indeed, in a high-dose sepsis model in mice, the HH/LC phenotypic variant was associated with accelerated death, although blood CFU levels were lower than observed with the LH/HC variant. We hypothesize that the accelerated death of mice infected with HH/LC isolates is a result of an overwhelming inflammatory response. The higher CFU levels of the LH/HC variant, on the other hand, may be associated with their increased resistance to phagocytic clearance, as comprehensively investigated in 4 different experimental models, i.e., whole blood, neutrophils and complement, intracellular survival in neutrophils, and blood collection in the mouse model. Because complete elimination of β-h/c by targeted mutagenesis is known to diminish GBS resistance to phagocytic killing (*16*) and to reduce blood survival in a variety of animal models (*10*,*12*,*14*,*16*), the results imply a simultaneous up-regulation of a factor with a more critical role in phagocyte resistance in the LH variant. The hyperencapsulation in the LH variant is in line with this reasoning, because the capsule is known to impair opsonophagocytosis (*4*) and likely contributes to the enhanced resistance of the LH phenotype in vitro and in vivo.

In summary, our data show that phenotypic variants with markedly different expression of prominent virulence factors can arise in the course of invasive GBS infection in humans. These bacterial subpopulations may contribute to different aspects of disease pathogenesis. In the case reported here, we could hypothesize that the HH/LC variant exerts toxin-mediated direct tissue injury and proinflammatory effects and that the LH/HC variant displays enhanced resistance to phagocytic clearance by virtue of increased capsule. The evolution of genetic switch mechanisms by the pathogen may allow it to conserve biochemical resources and synthesize the highest levels of capsule only under in vivo conditions of phagocyte selective pressure. The disadvantages of constitutive high-level capsule expression may lie in the known inhibitory effects of capsule on epithelial cell adherence (*38*) and the reduced growth rate of the LH/HC variant as observed in our studies in optimal culture media. The potential for GBS phenotype variants should be a consideration in the diagnostic microbiology laboratory and in future analysis of GBS molecular pathogenesis.
